# Unusual case of ATTR amyloidosis with cardiac manifestation and situs inversus totalis

**DOI:** 10.1007/s00392-016-1067-9

**Published:** 2017-01-23

**Authors:** Max Fritschka, Michael Schlegl, Adrian Borges, Mathias Werner, Rolf Gebker, Burkert Pieske, Sebastian Kelle

**Affiliations:** 1 Klinik für Innere Medizin mit Schwerpunkt Kardiologie, Campus Virchow-Klinikum, Charité-Universitätsmedizin Berlin, Augustenburger Platz 1, Berlin, 13353 Germany; 2 Klinik für Innere Medizin und Kardiologie, Deutsches Herzzentrum Berlin, Augustenburger Platz 1, 13353 Berlin, Germany; 3 DZHK (German Centre for Cardiovascular Research), Partner Site Berlin, 652133, Berlin, 13357 Germany; 4 Praxis Westend, Cardiology Outpatient Clinic, Spandauer Damm 130, 14050 Berlin, Germany; 5 HELIOS Klinikum Emil von Behring, Abteilung für Innere Medizin I / Kardiologie, Walterhöferstraße 11, Berlin, 14165 Germany; 6 HELIOS Klinikum Emil von Behring, Institut für Pathologie, Walterhöferstraße 11, Berlin, 14165 Germany

Sirs:

Dextrocardia is a rare condition and occurs in approximately 0.01% of the general population [1]. The incidence of amyloidosis is estimated at between 5 and 13 per million inhabitants per year [2]. Cardiac involvement occurs in primary (amyloid light-chain, AL) amyloidosis (40–50%), transthyretin (ATTR) amyloidosis (almost all cases) and in rare cases in secondary (amyloid A, AA) amyloidosis [3]. To our knowledge, the coincidence of situs inversus totalis and cardiac ATTR amyloidosis has never been reported before.

**Fig. 1:**
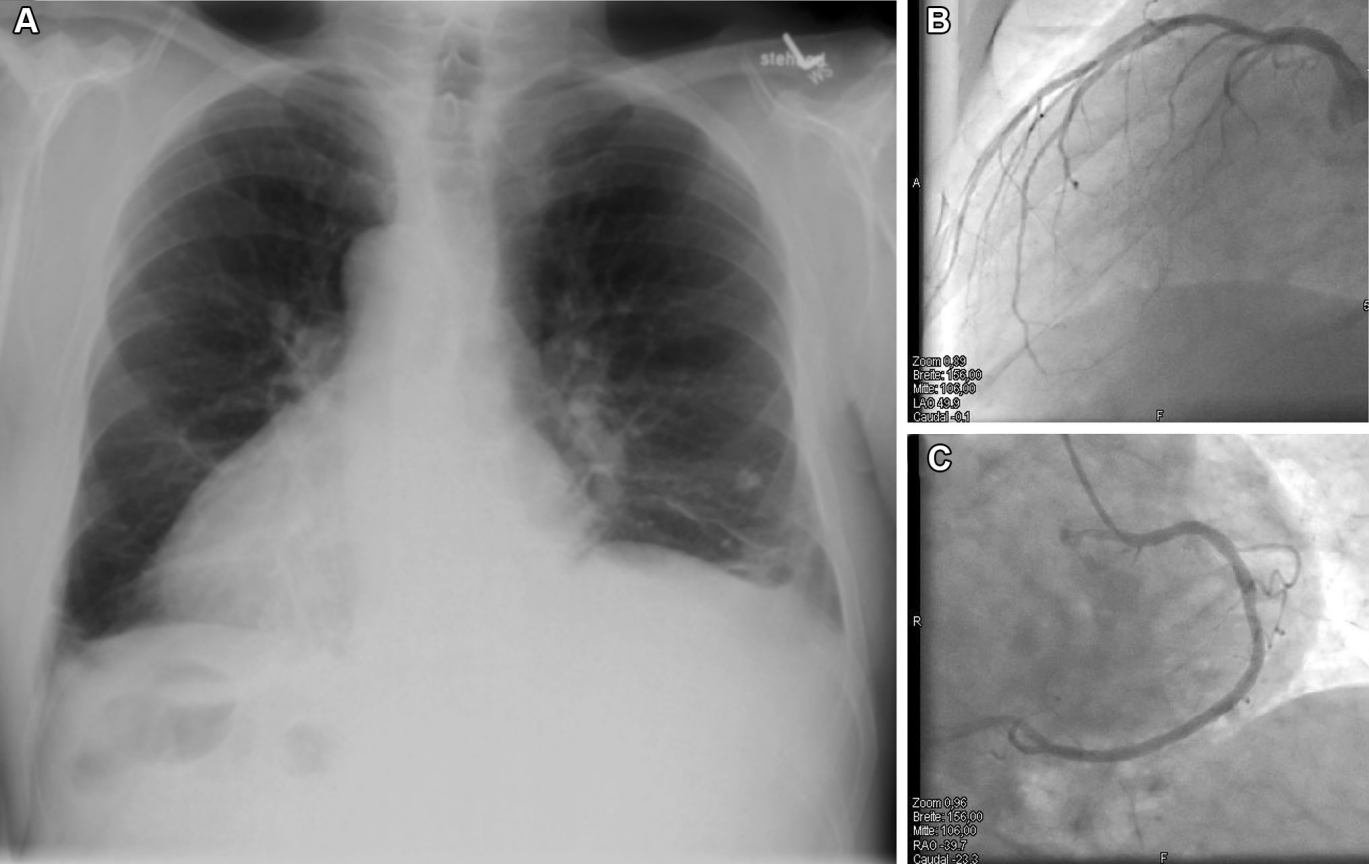
**a** The chest radiograph demonstrates a situs inversus totalis with dextrocardia and right-sided aortic arch. **b** Angiography of the left coronary artery (LAO 49.9). **c** Angiography of the right coronary artery (RAO −39.7)

**Fig. 2:**
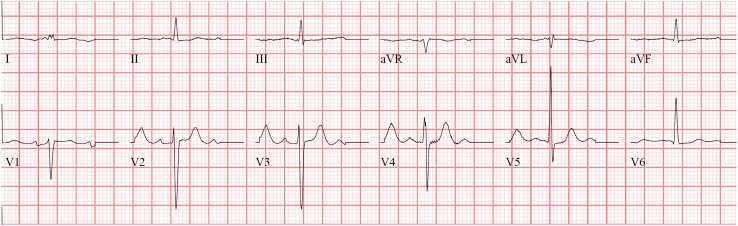
The electrocardiogram shows a sinus rhythm with low voltages in the limb leads (typical for cardiac amyloidosis would be all limb leads <5 mm in height). Further a typical slow R-progression as a pseudoinfarction pattern in the chest leads as well as a nonspecific intraventricular conduction delay is displayed

**Fig. 3:**
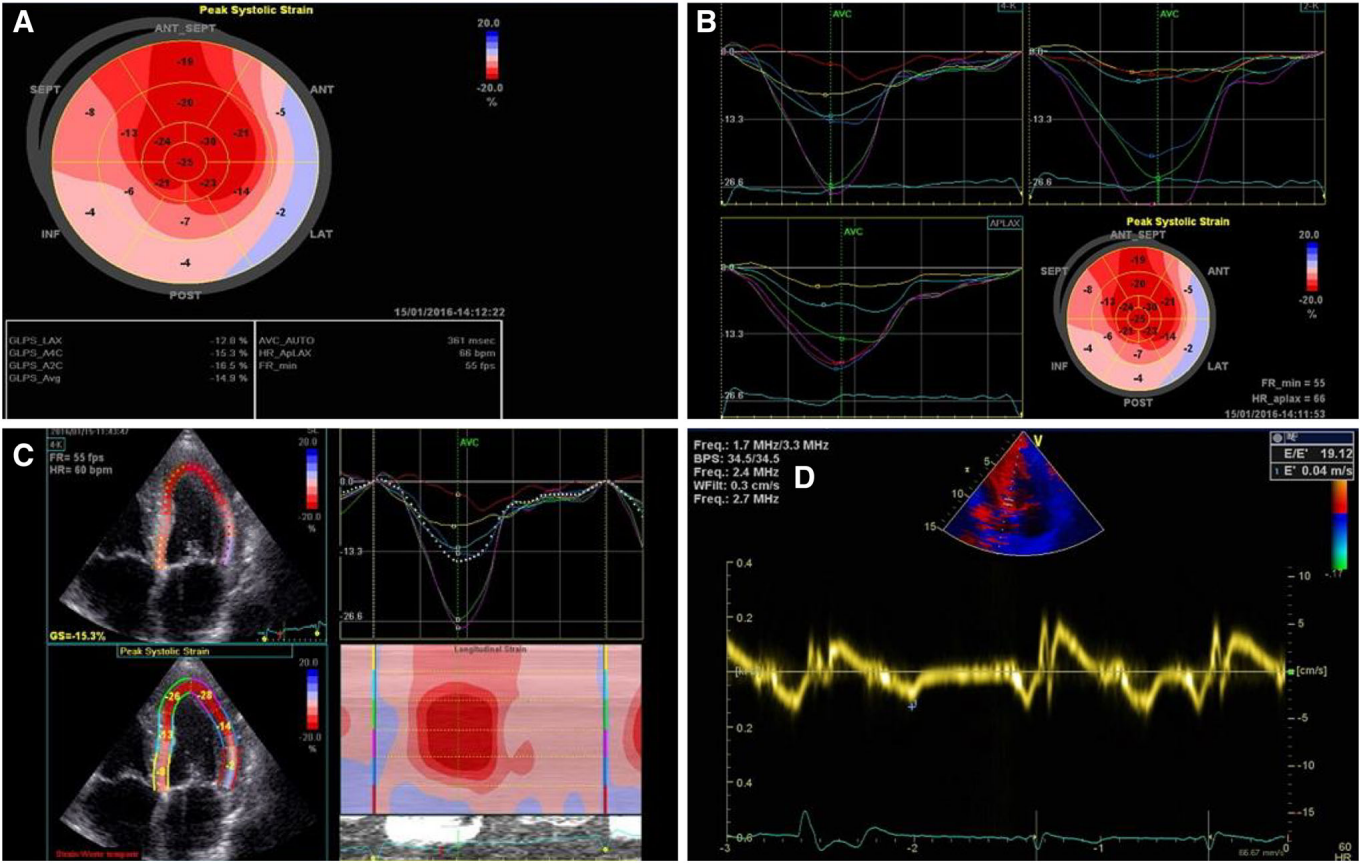
**a**–**c** 2D strain analysis with apical sparing as a typical sign for amyloidosis. **d** Spectral tissue Doppler (TDI) shows an antegrade systolic and two retrograde waves (*E*′ and *A*′). *E*/*e*′ measured 19.12 as an indication for a severe diastolic dysfunction

**Fig. 4:**
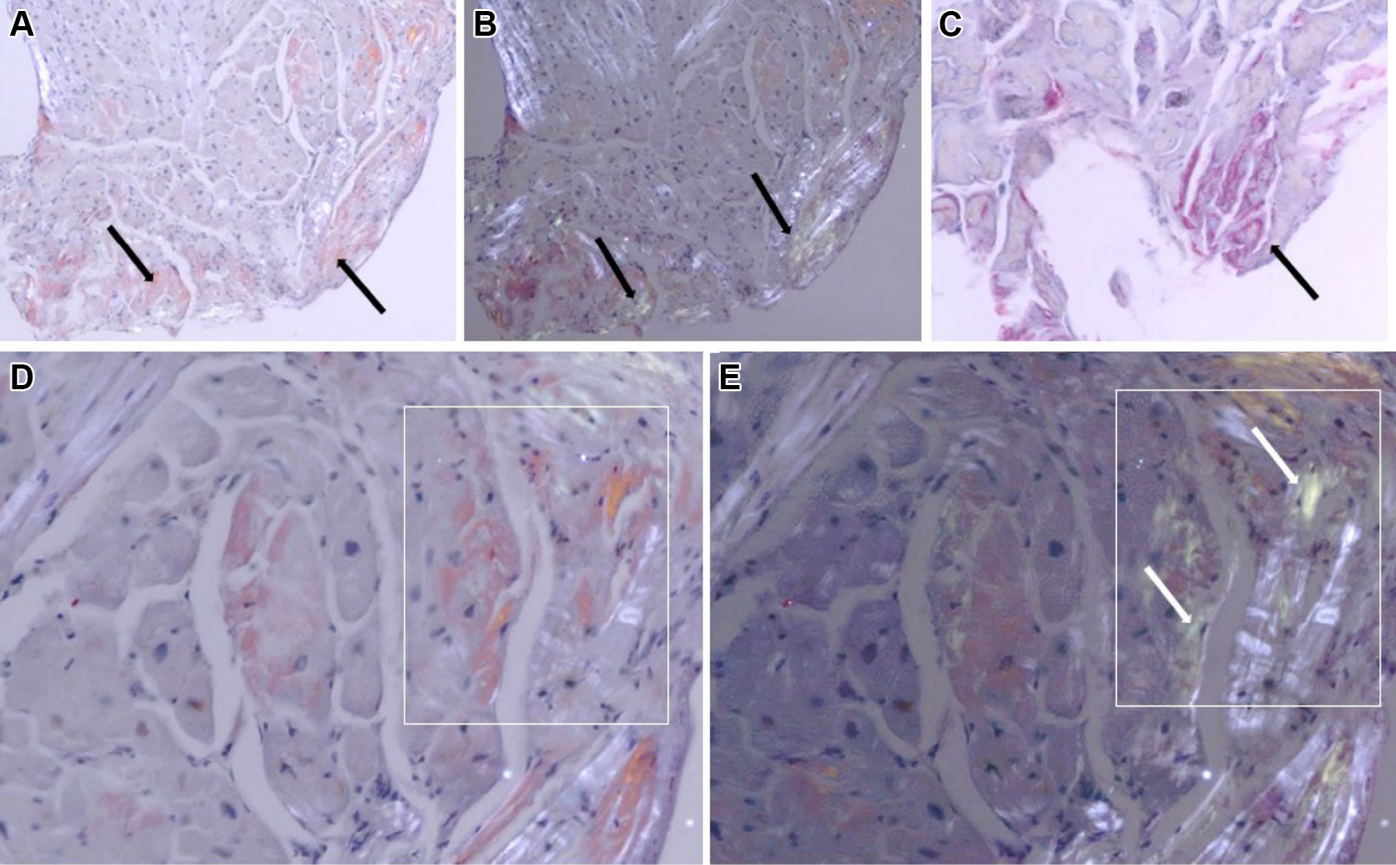
**a** Myocardial biopsy with diffusely spread congophilic material. **b** *Red* congophilic material turns *apple* *green* in *color* on polarizing light. **c** *Red* congophilic material marked by an ATTR antibody. **d** *Apple green* birefringence demonstrated by congophilic amyloid fibrils on polarizing light microscopy in cardiac biopsy specimen. **e** Collagen shows a shining *white* appearance

**Fig. 5:**
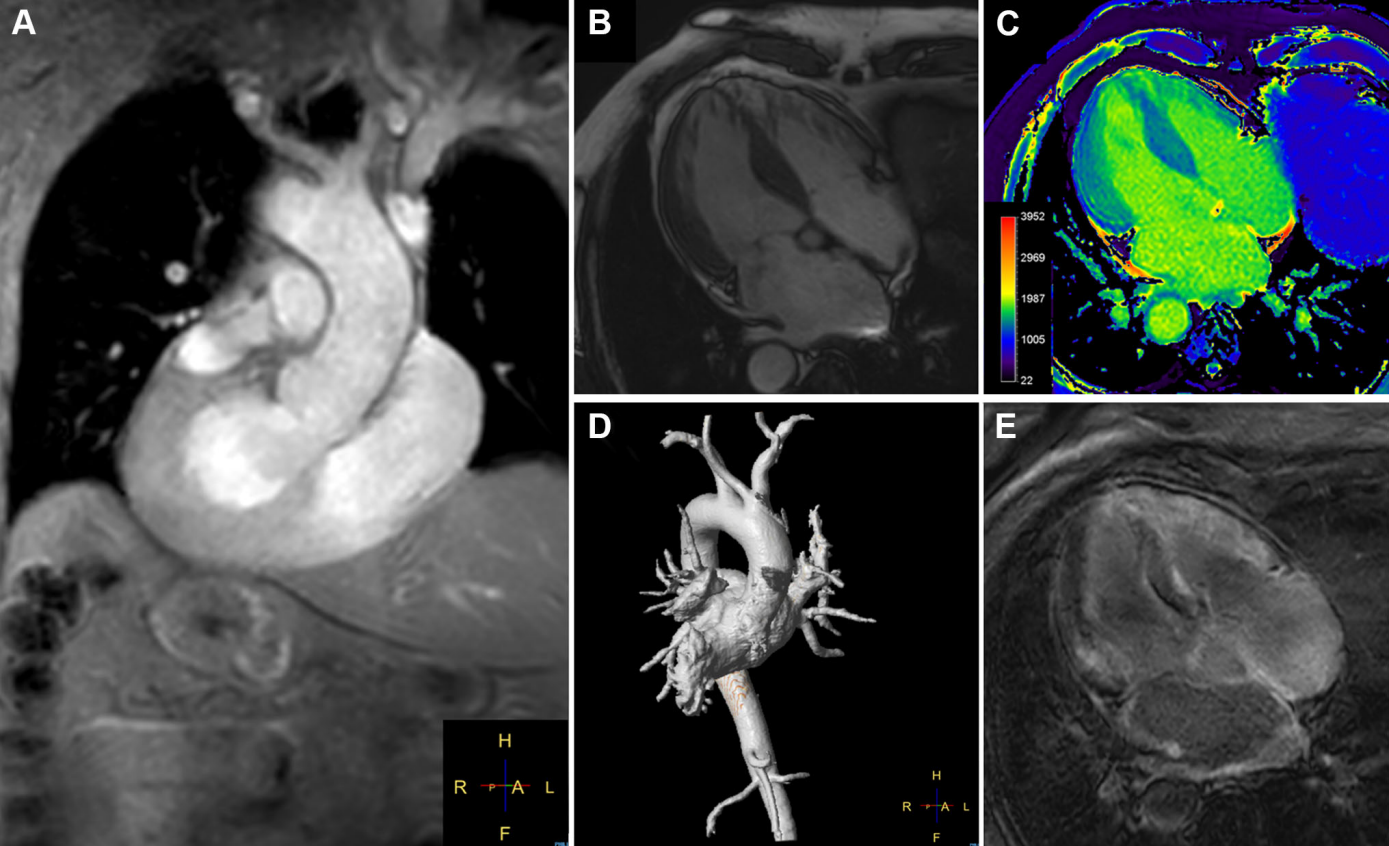
**a** Images from a cardiac MRI confirms a situs inversus with dextrocardia. The outflow tract of the left ventricle, the left atrium and the right-sided aortic arch are displayed. **b** Four-chamber view with the mirrored left and right ventricle and thickened left ventricular wall. **c** Contrast-free T1-mapping reveals elevated values for T1-times indicating increased amount of diffuse myocardial fibrosis. **d** Three-dimensional reconstruction shows the aorta and pulmonary trunk. **e** Late gadolinium enhancement of the left ventricle demonstrates high signal intensity patterns, typical for a clinical picture of cardiac amyloidosis

We are reporting the case of a 66-year-old male patient with known situs inversus totalis (Fig. 1a) and arterial hypertension who was hospitalized for right-sided angina pectoris with pain radiating into the neck and mandible alongside exertional dyspnea of NYHA class II. Laboratory results showed mildly positive troponin (high sensitivity): 24 ng/l; slightly elevated CK: 196 U/l; elevated NT-proBNP: 3496 ng/l; CKD stage III, creatinine: 1.07 mg/dl, GFR: 46 ml/min CKD-EPI. Urinalysis and other laboratory tests were without any significant pathological findings. The electrocardiogram showed sinus rhythm with low voltage in the limb leads (Fig. 2). A treadmill exercise test documented ventricular runs, triplets and multiple polymorphic extrasystoles under submaximal load without electrocardiographic ST-segment changes. The spirometry including body plethysmography and DLCO showed a restrictive ventilatory disturbance without pathological gas transfer. The transthoracic echocardiogram presented good left ventricular function (LVEF: 59% biplane Simpson), slight hypokinesis in septal basal and medial segments and severe diastolic dysfunction of grade III with intermediate hypertrophy (IVSd: 15 mm) (Fig. 3). The systolic pulmonary artery pressure was measured at 51 mmHg (+CVP) indicating pulmonary hypertension. No significant valvular heart disease was found. Relative apical sparing was seen in the 2D strain analysis which led to the suspicion of cardiac amyloidosis. Cardiac catheterization (Fig. 1b, c) ruled out a coronary artery disease and a left ventricular biopsy was conducted to obtain endomyocardial tissue samples to confirm the diagnosis (Fig. 4). The pathohistological results were positive for a cardiac manifestation of ATTR amyloidosis. Congophilic material was detected in the congo red dye, which could be marked immunohistochemically with transthyretin antibodies. Finally, cardiac MRI could demonstrate the known situs inversus totalis and diffuse myocardial fibrosis (elevated T1-times of around 1400 ms measured with a 3.0 Tesla clinical cardiac MRI scanner) of the inferolateral (medial/basal) left ventricle typical of amyloidosis (Fig. 5). In view of these results, medical therapy with tafamidis, doxycycline (off-label), ursodeoxycholic acid, green tea (off-label) [4], a beta-blocker and diuretics was started.

We report a rare case of ATTR amyloidosis with cardiac manifestation in a patient with situs inversus totalis. There seems to be no reported case of the coincidence of these two rare conditions to date. Yet cases of Kartagener syndrome (situs inversus, chronic sinusitis, and bronchiectasis due to primary ciliary dyskinesia) as an autosomal recessive disorder alongside renal amyloidosis are found in the current literature [5, 6]. Bronchiectasis is a known cause of secondary amyloidosis [7–9]. In this reported case no signs of proteinuria in the urine analysis or of bronchiectasis were found, with the spirometry showing a restrictive flow volume curve. Cardiac MRI, along with 2D strain evaluation conducted in transthoracic echocardiography or MRI [10] may lead to the non-invasive diagnosis of cardiac amyloidosis, with definite confirmation through endomyocardial biopsy. Yet a recent publication showed that today the diagnosis of ATTR amyloidosis could also be reached in patients without a monoclonal gammopathy using clinical information and bone scintigraphy alone [11], which might make a biopsy for diagnosis dispensable in certain cases in the future.
